# A measure to estimate the risk of imported COVID-19 cases and its application for evaluating travel-related control measures

**DOI:** 10.1038/s41598-022-13775-0

**Published:** 2022-06-09

**Authors:** Heewon Kang, Kyung-Duk Min, Seonghee Jeon, Ju-Yeun Lee, Sung-il Cho

**Affiliations:** 1 Institute of Health and Environment, Graduate School of Public Health, Seoul National University, Seoul, Republic of Korea; 2 Department of Public Health Science, Graduate School of Public Health, and Institute of Health and Environment, Seoul National University, 1 Gwanak-ro, Gwanak-gu, Seoul, 08826 Republic of Korea

**Keywords:** Infectious diseases, Epidemiology

## Abstract

High connectivity between nations facilitates the spread of infectious diseases. We introduce an improved measure to estimate the risk of COVID-19 importation. The measure was applied to identify the effectiveness of travel-related control measures. We estimated the risk of importation, using the product of air-travel volume and COVID-19 prevalence in the area-of-origin. Travel volumes were acquired through real-time mobile data, and prevalence was calculated considering the time-varying strength of the COVID-19 testing policy. With the measure, the number of expected-imported cases was calculated, and compared with the reported-imported COVID-19 cases before and after post-entry quarantine for all entrants. The expected and reported-imported cases were well fitted (R^2^ = 0.8). A maximum of 35 undetected-imported cases was estimated to have entered Seoul, before the first imported COVID-19 case was confirmed. With the travel-related control measures, at most, 48 (73%) imported cases could be isolated from the local community. Our measure predicted trends in imported COVID-19 cases well. The method used to develop the measure can be applied to future emerging infectious diseases. Our results provide a ‘real-world’ evidence that travel-related control measures are effective at curbing further COVID-19 transmission.

## Introduction

An outbreak of a novel coronavirus disease, now called COVID-19, was first identified in Wuhan, China, in December 2019. The disease spread quickly worldwide and more than 150 million cases were confirmed by May 2021^1^. As the disease was first reported in China, the first detection of COVID-19 in most countries outside China, including Korea, Japan, and the United States of America, was travel-associated^2^. International travel played a substantial role in the importation of COVID-19 into regions outside China. Local COVID-19 transmission was detected in nearly 50 nations by March 2020^3^.

Several travel-related control measures have been taken to reduce the risk of importation^4^. For example, Korea has conducted body temperature screening, health status surveys (presence of any COVID-19 related symptoms in the last 14-days), and received travel record declaration forms from all entrants since 19 March^5^. As the risk of travel-associated COVID-19 persisted and asymptomatic infections and transmission were repeatedly reported^6^, the Korean government, as those of other nations, implemented a 14-day post-entry quarantine for all entrants^5^. Seoul, the capital city with a population of 10 million, took more pre-emptive action by conducting polymerase chain reaction (PCR) tests on the day of arrival for all entrants who resided in Seoul. All entrants were subject to 14-day post-entry quarantine regardless of a negative test result.

The number of undetected-imported cases is of question, as it provides evidence regarding whether efforts to curb local transmission originating from imported cases are effective. The number of undetected-imported cases must also be considered when estimating the number of cases in the local community. Further, the extent of undetected-imported cases by time periods indicate whether there was a loophole in controlling travel-related cases. Particularly in the beginning of the epidemic, measures to control and prevent infections may have been limited due to evidence regarding COVID-19, and quarantine at the point-of-entry may not have been as effective as now.

Real-time travel volume, the numbers of cases in the source countries, and the ability to detect cases (i.e., surveillance capacity) should be considered in estimating the risk of travel-associated COVID-19. Travel volume is obviously related to imported cases, and the number of cases and surveillance capacity combined reflect the true size of the pandemic in the source countries^7^. Although several studies have estimated importation risks^8–14^, none of these used real-time travel data. They used either historical data from before importation risks were estimated^8–13^, or the number of airports connecting sites of interest^14^. Some of the early studies estimating imported cases from China did not consider the prevalence of COVID-19 in the source country^8,10,13^. Greater emphasis must be placed on these limitations, as travel volume may be inversely proportional to local prevalence due to a travel ban. Decreased external activities^15^ and travel volume^16^ following increases in new disease cases have been reported. As the COVID-19 outbreak developed into a pandemic, every country enacted some form of travel restrictions, and international tourist decreased significantly, throughout 2020–2021^17^. The extent of the decrease differed between continents^17^. Thus, studies using non-real-time travel data are unlikely to reflect real changes in passenger flow during the pandemic. Moreover, numerous studies have assumed a constant reporting rate, while testing policy strengths have changed markedly^8,10,11,13^. Here, we developed a new measure to estimate the risk of imported COVID-19 cases by multiplying travel volume derived from mobile data collected during the outbreak, and prevalence in the source country, which were derived considering the time-varying strength in the testing policy.

**Figure 1:**
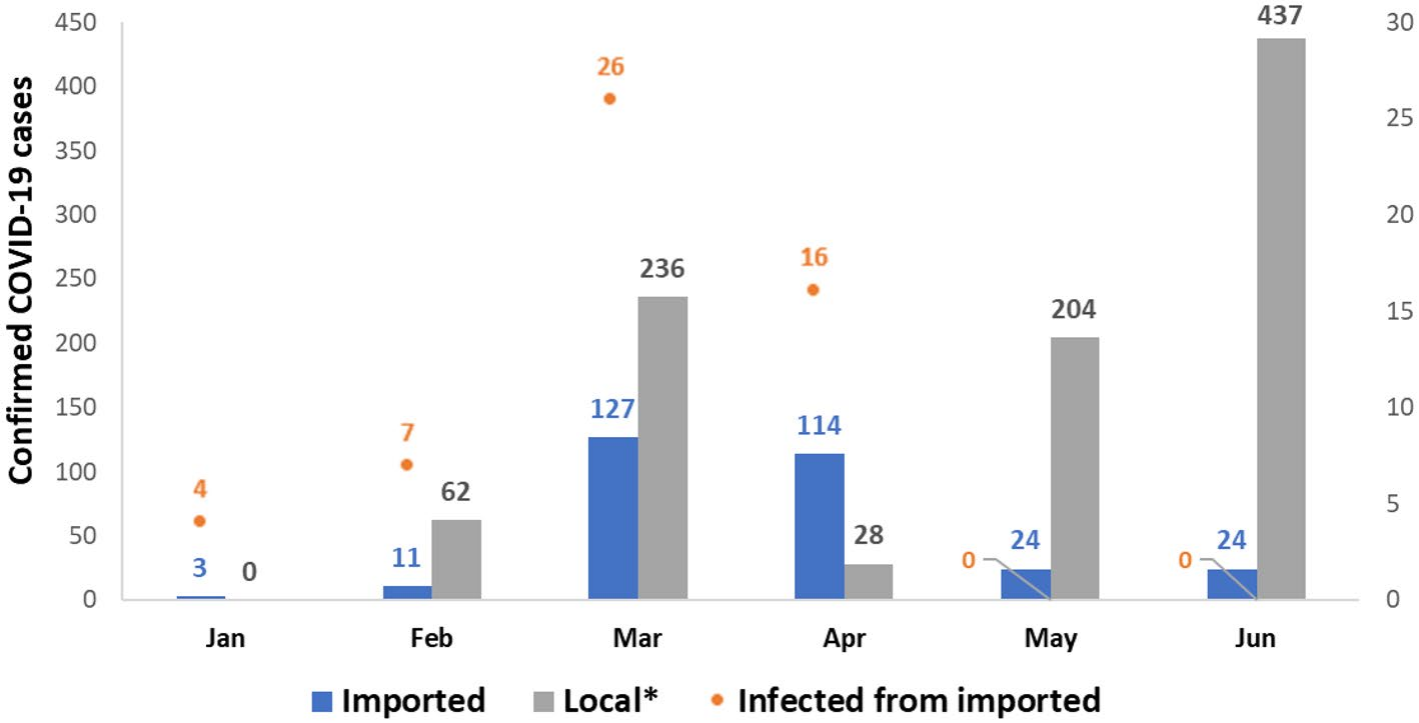
Imported, local COVID-19 cases and secondary cases from imported cases in Seoul, Korea (reconstructed based on the estimated infection source of the confirmed cases).

The number of imported COVID-19 cases in Seoul increased drastically in March compared to January and February of the same year (Fig. 1)^18^. However, the increases were relatively small during March and April, and zero secondary cases from imported cases were recorded during May and June, suggesting the measures to prevent transmission from imported cases have been highly effective. We assumed after the mandatory testing and post-entry quarantine that the detecting proficiency of COVID-19 would have been extremely high. Based on this assumption, we estimated the effectiveness of post-entry quarantine on the importation risk of COVID-19. Specifically, we fit a regression model of the number of imported COVID-19 cases after the policies came into full effect. Using the model, we computed the expected number of imported cases before the policies were implemented.

Further, using the developed measure, we estimated the reporting rate, and the number of avoided imported COVID-19 cases in Seoul as travel-related control measures (i.e., post-entry quarantine) were implemented. A recent Cochrane review of international travel-related control measures concluded that most of the evidence was acquired from modelling studies^19^. The authors pointed there was little ‘real-world’ evidence, particularly for measures including traveler quarantine. Seoul implemented mandatory testing for all arrivals since April. As the current study included both the periods before and after mandatory testing, it is suited for providing real-world evidence on the effectiveness of travel-related control measures.

## Results

### Entrants to Seoul and COVID-19 prevalence

**Figure 2:**
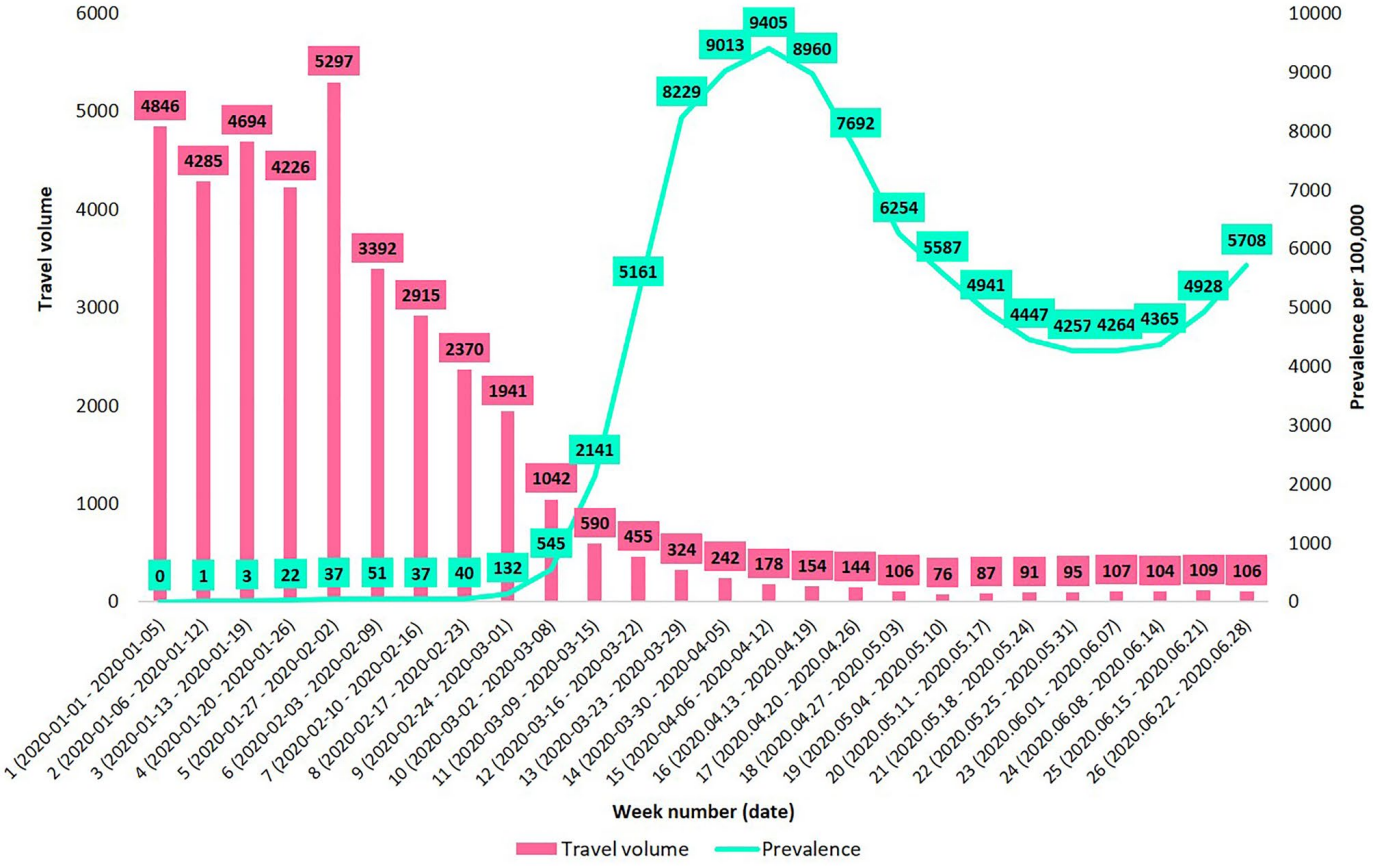
Average weekly travel volume to Seoul and average COVID-19 prevalence per 100,000 from the selected 30 countries.

The average weekly number of entrants to Seoul and the estimated COVID-19 prevalence per 100,000 from the selected 30 countries are presented in Fig. 2. The Supplementary File gives prevalence rates estimated with different reporting rate assumptions. The average weekly number of entrants from the selected countries to Seoul was 4846 during week 1 (1–5 January). The weekly travel volume increased to 5297 during week 5 (27 January–2 February), then decreased dramatically to 106 during week 26 (22–28 June). In contrast, the average local COVID-19 prevalence per 100,000 in the selected countries increased drastically from 0 during week 1 to 9405 during week 15 (6–12 April). The prevalence of COVID-19 decreased consecutively until week 22 (25–31 May), and then it rebounded to 5708 during week 26. Thus, the product of entrants to Seoul and COVID-19 prevalence in the source countries peaked during week 13, decreased to week 21, then increased again to week 26. The weekly average travel volume to Seoul and the COVID-19 prevalence per 100,000 for each country are provided in the Supplementary File (Figs. S1 and S2).

### Reported, expected, and undetected imported cases and the reporting rate

The measure yielded the weekly expected number of imported COVID-19 cases with the product of entrants and prevalence in the source country. The reported imported cases during the model fitting period (weeks 16–26) were all within the 95% confidence intervals of the expected imported cases (Fig. S3). The $R^{2}$ statistic of the observed and expected imported COVID-19 cases was 0.75, indicating that 75% of the variance in the imported cases is explained by the variance of the product of prevalence and travel volume. Table 1 depicts the weekly reported, expected, undetected imported cases along with the reporting rate of the imported COVID-19 cases before the policies came into effect. No imported cases were reported from the 30 countries during weeks 1–3. However, according to the model results, the total expected number of imported cases during weeks 1–3 was 13.2 (95% CI 1–35). The estimated number of undetected imported cases was highest during week 10 (2–8 March) at 5.8 (95% CI 1–11). The total number of undetected imported cases between 1 January and 29 March was estimated to be 41.6 (95% CI 3–177). The reporting rate was generally low during January and February. However, it improved to 100% during week 12 (16–22 March) (95% CI = 36.7–100) and week 13 (23–29 March) (95% CI 69.6–100). The average reporting rate during this period was 37.2% (95% CI 19.2–76.9%).

**Table 1:** Weekly reported, expected, undetected imported cases, and reporting rate.

Week number (Date)	Reported imported cases^a^	Expected imported cases (95% CI)	Undetected imported cases^b^ (95% CI)	Reporting rate (%)^c^ (95% CI)
1 (2020-01-01–2020-01-05)	0	4.3 (0–12)	4.3 (0–12)	0 (0–100)
2 (2020-01-06–2020-01-12)	0	4.4 (0–11)	4.4 (0–11)	0 (0–100)
3 (2020-01-13–2020-01-19)	0	4.5 (1–12)	4.5 (1–12)	0 (0–0)
4 (2020-01-20–2020-01-26)	1	4.9 (1–11)	3.9 (0–10)	20.3 (9.1–100)
5 (2020-01-27–2020-02-02)	3	6.5 (1–12)	3.5 (0–9)	46.0 (25.0–100)
6 (2020-02-03–2020-02-09)	3	6.0 (1–11)	3.0 (0–8)	50.4 (27.3–100)
7 (2020-02-10–2020-02-16)	0	5.1 (1–11)	5.1 (1–11)	0 (0–0)
8 (2020-02-17–2020-02-23)	1	4.7 (1–12)	3.7 (0–11)	21.3 (8.3–100)
9 (2020-02-24–2020-03-01)	4	5.1 (1–10)	1.1 (0–6)	78.0 (40.0–100)
10 (2020-03-02–2020-03-08)	0	5.8 (1–11)	5.8 (1–11)	0 (0–0)
11 (2020-03-09–2020-03-15)	5	7.4 (1–15)	2.4 (0–10)	68.0 (33.3–100)
12 (2020-03-16–2020-03-22)	22	11.5 (1–60)	0.0 (0–38)	100 (36.7–100)
13 (2020-03-23–2020-03-29)	64	13.2 (0–92)	0.0 (0–28)	100 (69.6–100)
Total/Average^d^	103	83.3 (10–280)	41.6 (3–177)	37.2 (19.2–76.9)

^a^Indicates cases from the selected 23 countries.

^b^Expected imported cases – reported imported cases.

^c^Expected imported cases/reported imported cases * 100.

^d^The total is provided for the reported, expected, undetected imported cases, and the average is provided for the reporting rate.

### Effectiveness of post-entry quarantine

We calculated the number of avoided imported cases between April and June using the estimated reporting rate of 37.2% (95% CI 19.2–76.9%) if mandatory testing on the day of arrival and post-entry quarantine had not come into effect (Table 2). The expected number of identifiable imported COVID-19 cases was 18 under the reporting rate assumption of 19.2% and 58 cases under the reporting rate assumption of 76.9%, whereas the reported number of imported cases was 66. As Seoul has had mandated testing and quarantine since 1 April, a minimum of eight to a maximum of 48 imported cases were isolated and avoided from spreading COVID-19 in the community between 20 April and 28 June.

**Table 2:** Estimated identifiable and avoided imported cases at different reporting rate assumptions.

Week number (date)	Reporting rate			
	19.2%	37.2%	76.9%	100% (Reported cases)
16 (2020.04.13–2020.04.19)	3	5	9	11
17 (2020.04.20–2020.04.26)	1	2	4	4
18 (2020.04.27–2020.05.03)	2	3	7	8
19 (2020.05.04–2020.05.10)	1	1	2	2
20 (2020.05.11–2020.05.17)	1	2	3	3
21 (2020.05.18–2020.05.24)	2	3	7	8
22 (2020.05.25–2020.05.31)	2	3	7	8
23 (2020.06.01–2020.06.07)	1	2	4	4
24 (2020.06.08–2020.06.14)	1	2	4	5
25 (2020.06.15–2020.06.21)	2	3	5	6
26 (2020.06.22–2020.06.28)	2	3	6	7
Total identifiable	18	29	58	66
Cases avoided^a^	48 (72.7%)	37 (56.1%)	8 (12.1%)	–

^a^Cases avoided = Total number of reported imported cases − Total number of expected imported cases for each reporting rate assumption.

## Discussion

We developed a new measure to estimate the risk of imported COVID-19 cases that is calculated using the product of air travel volume and local COVID-19 prevalence. Using the measure, the number of imported infectious diseases can be estimated, and the efficacy of travel-related control measures can be evaluated. Compared to previous models, we considered COVID-19 prevalence in the source country, time-variant strength of the testing policy, and fitted the model to a location and period where the detection capacity was very efficient. We found that many undetected-imported cases entered Seoul, well before the first confirmed case of neither contact history to a confirmed case nor travel-history was identified, and even before the very first confirmed case in Seoul. Travel-related control measures, specifically mandatory testing on the day of arrival and post-entry quarantine in Seoul, Korea, was effective for preventing the local introduction of the disease. The reporting rate of imported COVID-19 cases in Seoul increased over time.

Our measure of importation risk predicted the trends in imported COVID-19 cases well. The model well explained the observed imported cases. The measure we developed is highly suitable to estimate the risk of imported COVID-19 cases or any future emerging infectious disease risks. The majority of previous studies did not use COVID-19 prevalence in the source location to estimate the number of imported cases^8–10,13,14^. These methods may be convenient to estimate the risk of imported cases from one country (China, in the above cases) to other countries. However, as an outbreak from a single epicenter proceeds to a global pandemic, such a measure may no longer be useful, as different risks of exposure in different source countries cannot be considered. Furthermore, a measure suitable to estimate the number of imported cases from various locations in a single country is much more useful, as responses to imported infectious diseases are required most at the country-level. Even those that had considered the number of COVID-19 cases in the source countries used historical air travel data to estimate travel volume. As international travel underwent an unprecedented decrease during the pandemic^17^, it is unreasonable to use historical travel data to estimate imported cases, when 900 million fewer international arrivals were reported during January–October 2020. The extent of decreases also differed by continents^17^.

Another important aspect of the measure we developed is that we considered the daily strength of the testing policy to derive the time-varying reporting rate, and thus handled the under-estimation of COVID-19 prevalence in the source country to an extent. While previous studies estimated the number of imported cases over less than 2 months, it is unreasonable to assume that detection capacity would remain constant if the study period were to be lengthened. Using the data provided by OxCGRT, we derived day-to-day reporting rates of imported cases by location. Future studies should use the time-varying reporting rate to derive estimates of COVID-19 prevalence.

Furthermore, we fit our model to a region and time in which almost all imported cases were identified. As a substantial proportion of COVID-19 cases are non-severe or asymptomatic, reported COVID-19 cases must always be assumed to be under-ascertained^20^. Niehaus et al. considered Singapore as having the greatest capacity for detecting imported cases and calculated the detecting capacity of countries relative to Singapore^13^. Yet, the imported cases derived from that study were described as lower-bound, as Singapore’s detection capacity may not be 100% efficient, and detection capacity that relies on symptoms and travel history is limited. In contrast, Seoul does not rely on the presence of symptoms or travel history to test for COVID-19 and has conducted PCR testing of entrants universally since 1 April, 2020. Thus, the ascertainment rate of the imported COVID-19 cases after the testing policy came fully into effect was assumed to be 100%, and our model dealt with the limitations of the previous models in which the estimates of imported COVID-19 cases had larger uncertainties.

The very first COVID-19 case in Seoul (of course, an imported case) was identified on the 24th of January 2020. Yet, our model estimates suggested at least one and up to a maximum of 35 imported COVID-19 cases entered Seoul before the first imported case was identified. Further, the model results suggest that before an 82-year-old case which was the first case with neither contact nor travel history was confirmed on the 16th of February 2020, a maximum of 62 imported COVID-19 cases entered Seoul freely. By April, almost 200 undetected-imported cases were estimated to have entered Seoul. These undetected-imported cases entering the local community may have led to multiple cluster outbreaks including those in a hospital during February, and a call center during March.

Our average reporting rate of imported COVID-19 cases before some of the travel-related control measures came in effect (i.e. before March) was lower than previously reported rates of 38%^13^ or 25–30%^8^. The lower reporting rate may have resulted because we fit our model to a period when detection efficiency was very high. The model estimates suggested the reported imported cases for each week were all smaller or within the bounds of the expected imported cases. Although the reported imported cases for each week were smaller than the upper bound of the expected imported cases, we found that the model somewhat underestimated the imported cases during weeks 12 and 13. Consequently, the reporting rate increased over time, particularly in March. This may be due to the strengthened central-government measures. Entrants from all nations were subject to travel-related control measures beginning week 12. For example, temperature screening, health status questionnaires, and travel history declarations were required among entrants from China, Hong Kong, and Macau during February. The measures were expanded to Japan, Italy, Iran, and all of Europe by mid-March. Then, it was expanded to the entire world on 19 March^5^. Regardless of their nationality, all arrivals in Korea were required to verify their local address and contact information, install an app to monitor the presence of any symptoms regarding COVID-19, and adherence to quarantine. Thus, unlike other study periods, excess imported cases may have been identified, leading the model to underestimate imported cases during weeks 12–13.

The model estimates showed at least 8 and at most 48 imported COVID-19 cases could have been introduced into the community in 2 months without the 14-day post-entry quarantine for all entrants residing in Seoul, causing a loss of the transmission chain. This corroborates a former study reporting that testing and 14-day quarantine are highly effective for curbing local transmission^21^. The effective reproductive number^22^ began to rise at the end of April, reached > 2 in May, then decreased gradually to 1 during June 2020^23^. Given that the effective reproductive number exceeded 1 during late April and June, secondary cases could have been averted due to the preventive measures for the importation risk. The number of secondary cases that would have arisen from the undetected imported cases before April remains to be determined.

The study limitations should be discussed. First, some of the infected but still-in-the-latent-period individuals may not have tested positive on the day of arrival. However, 80% of infected travelers are reported to be infectious upon arrival^21^. During the quarantine period, the entrants were required to report any symptoms regarding COVID-19 using an app and were tested again for the presence of any symptoms. As our estimates were calculated every week, and the median incubation period is 5.1 days^24^, we assumed that most cases were included within a week of confirmation. Second, we arbitrarily assigned the strength of the testing policy to the point estimate and the CI of the previously suggested reporting rate^24^. However, the true reporting rate of these testing strengths is unknown, and results based on different reporting rates were not materially different. Third, although we assumed a detection rate of 100%, PCR tests have a sensitivity of 97%^25^. A detection rate under 100% would mean more undetected cases and lower true effectiveness of travel-control related measures than we had estimated. However, as a 14-day quarantine, and symptom reporting were required regardless of the test results, the under-ascertainment would have been small. Yet, as even a few unidentified infections can compromise the effectiveness of travel-related control measures, adherence to quarantine and social distancing must be strict.

We developed a new measure to estimate the risk of imported COVID-19 cases. Using this measure, testing on arrival and post-entry quarantine were effective for preventing subsequent local transmission. We recommend the use of this measure to estimate the number of imported COVID-19 cases or any that of any future emerging infectious disease. In addition, the measure, along with the effective reproductive number, can be used to estimate the number of COVID-19 cases in a community.

## Methods

### Overall framework

**Figure 3:**
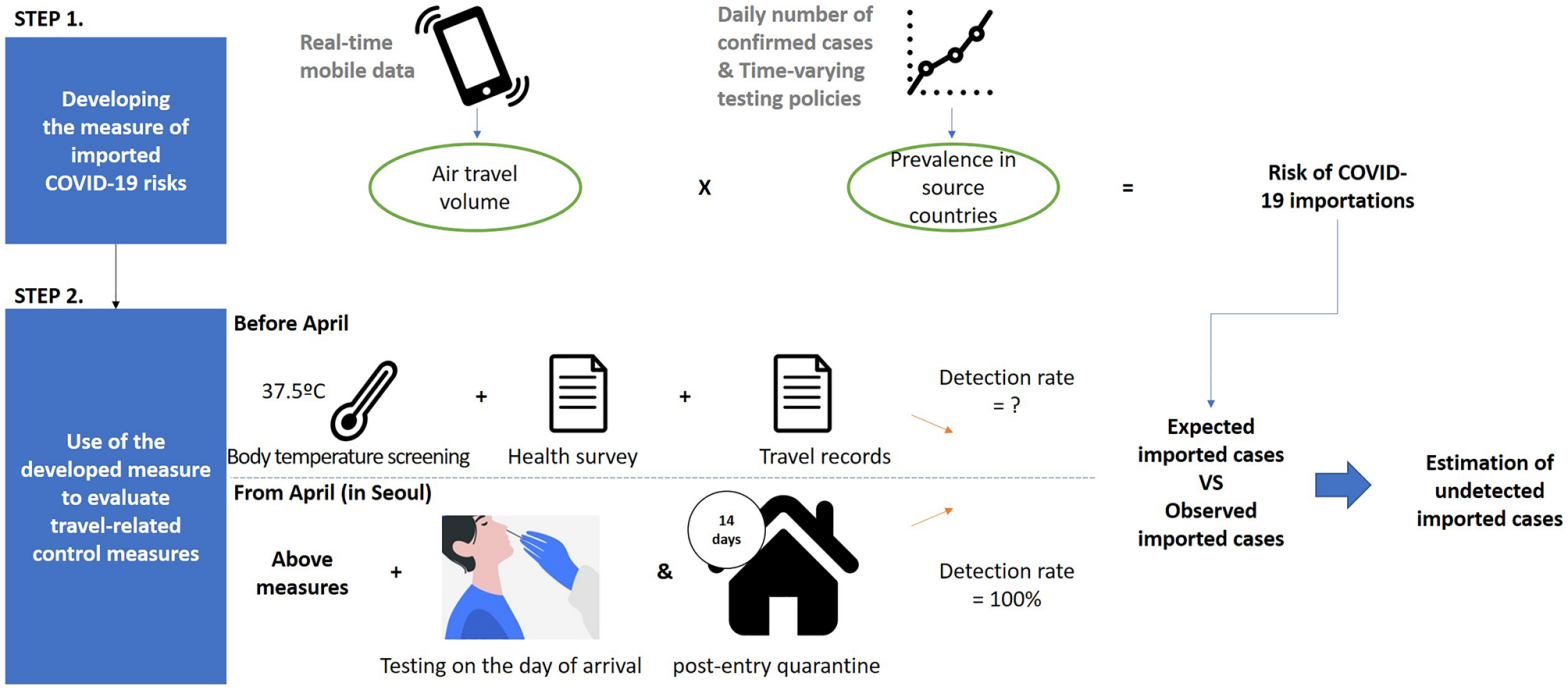
The methodological framework of the study.

Figure 3 outlines the methodological framework of the study. First, we developed a measure of imported COVID-19 risk. The rationale for the measure is the risk of importation is proportional to the number of entrants, and the prevalence of COVID-19 in the country of departure. Thus, air travel volumes from selected countries to Seoul and the prevalence of COVID-19 cases in the countries of departure were multiplied to derive the measure. The main improvement in the measure from the previous studies is that real-time mobile data were used to estimate air travel volume, and time-varying detection rates were considered to estimate the prevalence of COVID-19. Use of air travel data from the previous year^10^ and not considering the detection efficiency^13^ have been suggested as limitations in former studies.

Second, the developed measure was used to evaluate the travel-related control measures. Using the measure, we fit our model to the period when the detection rate was assumed to be 100%, as testing-on the day of arrival, and post-entry quarantine for 14 days were in effect. Then, we calculated the number of expected imported cases during the period when only body temperature screening, health surveys, and the declaration of travel records were required. The expected number was compared with the observed number of imported cases to estimate the number of undetected imported cases, and the detection rate before mandatory testing.

### Data sources

Data were obtained from three sources. First, the daily numbers of confirmed COVID-19 cases in Seoul were obtained^18^ to identify the number of imported COVID-19 cases in Seoul for the period between 24 January and 30 June, 2020, since the earliest COVID-19 case in Seoul was confirmed on 24 January 2020. Second, the daily numbers of roaming users from each country to Seoul between 1 January and 30 June were acquired from Korea Telecom (KT). Travelers from Korea to other countries use roaming services to make/receive calls from regions outside the coverage areas of their home networks. KT has the second largest market share among mobile operators in Korea at 31%^26^; therefore the KT data are sufficient to estimate the trend in the number of entrants to Korea. Third, the daily numbers of confirmed COVID-19 cases between 1 January and 7 July, were used^1^ to estimate the prevalence of COVID-19 in countries outside Korea.

### Dataset construction

#### Country selection

Countries of interest were selected based on the travel history of the reported imported COVID-19 cases in Seoul. For example, if an identified imported case had travelled to Italy, then Italy was selected. Cases with travel histories to more than a single country (24 cases) and unknown regions (two cases) were excluded from this procedure, as the source of infection could not be specified. Thirty countries were selected (Table 3). However, Austria, China, Malaysia, Poland, Singapore, Thailand, and Vietnam were excluded from the model fitting procedure as there were no reported imported cases from these countries after 1 April, 2020, when testing on the day of arrival and 14-day post-entry quarantine for entrants became mandatory. Including countries with no imported cases after the implementation of mandatory testing may introduce bias to the model estimates.

**Table 3:** Countries selected as eligible for model fitting.

Continent (number of countries)	Country
Americas (4)	Brazil, Canada, Mexico, and the United States of America
Asia (11)	Bangladesh, *China,* India, Indonesia, Iraq, Japan, Kazakhstan, Kyrgyzstan, *Malaysia,* Pakistan, Philippines, *Singapore, Thailand*, Turkey, the United Arab Emirates, *Vietnam*
Europe (8)	*Austria,* France, Germany, Ireland, Italy, *Poland,* Portugal, Russia, Spain, United Kingdom

The seven countries in Italic were excluded from the model fitting procedure as there were no reported imported cases from these countries after 1 April, 2020.

#### Entrants from each country to Seoul

The number of entrants from the selected countries to Seoul was calculated using the data provided by KT. These data provided the daily number of roaming users by country of departure and residential region in Korea during 2020. As we used the airline travel volume from a single mobile operator, the data do not represent the exact travel volume. Yet these data have been reported to be representative of the trends in domestic^27^, and international travel volume^28^.

#### Estimating the prevalence of COVID-19

The prevalence of COVID-19 in the selected countries were estimated to assess the risk of exposure to COVID-19 among entrants traveling to Seoul, Korea. The local prevalence of COVID-19 in the selected countries were derived based on the daily number of confirmed new cases^1^. The reported incidence of COVID-19 is considered to be underestimated due to incomplete testing^29,30^. Thus, we extended a method used previously^11^. The daily strength of the testing policy for each country was derived using the Oxford COVID-19 Government Response Tracker (OxCGRT)^31^. The OxCGRT classifies the strength of testing policy as follows: 0-no testing policy, 1-those who have both symptoms and meet specific criteria (key workers, classified as contacts, traveled overseas), 2-anyone showing symptoms, and 3-open public testing. Based on the reporting rate previously suggested: 0.092 (95% confidence interval [CI] 0.05, 0.20)^32^, we assigned testing policies 1, 2, and 3 to the reporting rates of 0.05, 0.092, and 0.20 respectively. The reporting rate for no testing policy (0) was assumed to be 0.01. The detection rates of two additional studies were considered as a sensitivity analysis. The results are provided in the Supplementary File.

The methods for estimating the incident ($$I_{t} )$$ and prevalent ($$P_{t} )$$ infectious cases on day *t*, considering the ascertainment period and reporting rate, is described in detail by Fauver et al.^11^ The model used prevalent cases as existing (prevalent) cases as well as new (incident) cases to serve as sources of infection. Briefly, $$I_{t - d - 2}$$ was estimated using the reported new cases ($$C_{t} )$$ and the reporting rate ($${\uprho }_{t} )$$ on day *t*. Time from symptom onset to isolation (testing) *d* was assumed to be 5 days, and cases were assumed to become infectious 2 days before symptom onset^33^.

1 $$I_{t - d - 2} = \frac{{C_{t} }}{{\rho_{t} }}$$

Then, $I_{t}$ and the probability that a patient who became infectious on day *i* was still infectious on day *t* was added to estimate $P_{t}$.

2 $$P_{t} = \mathop \sum \limits_{i = 1}^{t - 1} I_{i} \left( {1 - \gamma \left( {t - i} \right)} \right) + I_{t}$$

The cumulative distribution function $\left( {f\left( x \right)} \right)$ of the infectious period $\gamma \left( {t - i} \right)$ was assumed to follow a gamma distribution with a mean and standard deviation of 7 and 4.5 days, respectively. As Fauver et al. show, the shape (${\upalpha })$ and rate ($1/{\uptheta })$ of the gamma distribution was calculated^34^.

3 $$f\left( x \right) = \frac{{{\uptheta }^{{\upalpha }} }}{{{\Gamma }\left( \alpha \right)}}x^{{{\upalpha } - 1}} e^{{\frac{ - z}{\beta }}}$$

where $${\Gamma }\left( \alpha \right) = \mathop \smallint \limits_{0}^{\infty } t^{\alpha - 1} e^{t} dt$$.

The calculated $P_{t}$ was divided by the total population of each country in 2020 to estimate prevalence per 100,000. The datasets of the entrants and the COVID-19 prevalence were merged according to country and date. Weekly average entrant volume and COVID-19 prevalence per 100,000 were calculated using the merged dataset. Finally, the weekly sum of reported imported COVID-19 cases in Seoul was merged to the dataset containing the weekly average number of entrants and the COVID-19 prevalence.

### Statistical analyses

#### Description of the new measure

The method used by de Salazar et al.^10^ was extended to estimate the number of expected imported COVID-19 cases. The measure indicating the risk of COVID-19 importation was calculated as the product of the number of entrants and COVID-19 prevalence in the selected countries. Specifically, the expected number of imported cases was assumed to follow an over-dispersed Poisson distribution and was assumed to be dependent on the product of the entrants ($E_{w}$) and the prevalence per 100,000 on week* w* ($P_{w}$).

4 $$\begin{gathered} {\text{Expected number of imported cases}} = {\text{Quassipoisson}}\left( {\lambda_{w} } \right) \hfill \\ \lambda_{w} = \beta_{0} + {\upbeta }\left( {E_{w} \times P_{w} } \right) \hfill \\ \end{gathered}$$

#### Estimating the effectiveness of post-entry quarantine

The model was first fit conservatively based on the data from week 16 (13 April) onwards. Testing on the day of arrival and after the 14-day post-entry quarantine came into effect for all arrivals beginning on 1 April. However, as the median incubation period is 5.1 days and the 95% percentile is 11.7 days^35^, many reported imported cases during weeks 14 (2020.03.30–2020.04.05) and 15 (2020.04.07–2020.04.12) could have arrived before 1 April. The regression coefficient ${\upbeta }$ was estimated based on the data of week 16 and onwards using the maximum likelihood method. Then, the expected number of imported cases was calculated based on the estimated β. A bootstrap sample of 500,000 was used to compute the 95% CI for the expected number of imported cases. The model fit was assessed by identifying whether the reported imported cases were within the confidence intervals of the fitted estimates, and by using the $R^{2}$ statistic.

All results are provided by week number. We used data from 1 January to 30 June and the corresponding week (weeks 1–26), as the dates are provided in the tables. The number of undetected imported cases was computed by subtracting the number of reported imported cases from the number of expected cases. As in a previous study, upper or lower bounds for the undetected imported cases were calculated by subtracting the reported cases from the upper or lower bound of the expected imported cases^36^. The undetected cases were presented as 0 if the point estimate or CI of the undetected cases was < 0. Moreover, the reporting rate for the imported cases was computed as a ratio of reported imported cases to expected imported cases.

5 $${\text{Reporting }}\;{\text{rate }}\left( {\text{\% }} \right) = \frac{Reported\; imported \;COVID - 19\; cases}{{Expected\; imported \;COVID - 19\; cases}} \times 100$$

The lower bound for the reporting rate was computed as the ratio of the reported imported cases to the expected upper bound, and the upper bound for the reporting rate was calculated as the ratio of the reported imported cases to the expected lower bound. The reporting rate for undetected cases was presented as 100% if the reported cases exceeded the expected cases. By multiplying the calculated reporting rate and the reported imported cases after the testing and post-entry quarantine policies on the entrants came fully into effect, the number of avoided imported cases without these policies was computed.

### Ethical statement

The data used were either publicly available, completely non-identifiable data collected for the purpose of disease control, or in an aggregated form. Thus, no ethics approval was required.

## Supplementary Information

Supplementary Information.

## Data Availability

Number of imported cases in Seoul (https://www.seoul.go.kr/coronaV/coronaStatus.do), and the number of newly confirmed cases in each country (https://covid19.who.int) are publicly available. Roaming data from KT are available under the approval of the Korea Data Agency.

## References

[1] World Health Organization. *WHO Coronavirus Disease (COVID-19) Dashboard*. https://covid19.who.int/ (2020).

[2] World Health Organization (2020). Novel Coronavirus (2019-nCoV): Situation Report.

[3] World Health Organization (2020). Coronavirus Disease 2019 (COVID-19): Situation Report.

[4] Kang S (2020). The Evolving policy debate on border closure in Korea. J. Prev. Med. Public Health.

[5] Ministry of Health and Welfare. *COVID-19 Response: Preventing the Inflow and Spread of the Virus*, http://ncov.mohw.go.kr/en/baroView.do?brdId=11&brdGubun=111 (2020).

[6] Kronbichler A (2020). Asymptomatic patients as a source of COVID-19 infections: A systematic review and meta-analysis. Int. J. Infect. Dis..

[7] Liebig J, Najeebullah K, Jurdak R, Shoghri AE, Paini D (2021). Should international borders re-open? The impact of travel restrictions on COVID-19 importation risk. BMC Public Health.

[8] Bhatia S (2020). Estimating the number of undetected COVID-19 cases among travellers from mainland China. Wellcome Open Res..

[9] Chinazzi M (2020). The effect of travel restrictions on the spread of the 2019 novel coronavirus (COVID-19) outbreak. Science.

[10] De Salazar PM, Niehus R, Taylor A, Buckee COF, Lipsitch M (2020). Identifying locations with possible undetected imported severe acute respiratory syndrome coronavirus 2 cases by using importation predictions. Emerg. Infect. Dis..

[11] Fauver JR (2020). Coast-to-coast spread of SARS-CoV-2 during the early epidemic in the United States. Cell.

[12] Liebig J, Najeebullah K, Jurdak R, Shoghri AE, Paini D (2020). Should international borders re-open? The impact of travel restrictions on COVID-19 importation risk. BMC Public Health.

[13] Niehus R, De Salazar PM, Taylor AR, Lipsitch M (2020). Using observational data to quantify bias of traveller-derived COVID-19 prevalence estimates in Wuhan, China. Lancet Infect. Dis..

[14] Wells CR (2020). Impact of international travel and border control measures on the global spread of the novel 2019 coronavirus outbreak. Proc. Natl. Acad. Sci. USA.

[15] Slimani M, Paravlic A, Mbarek F, Bragazzi NL, Tod D (2020). The relationship between physical activity and quality of life during the confinement induced by COVID-19 outbreak: A pilot study in Tunisia. Front. Psychol..

[16] Lee H, Noh E, Jeon H, Nam EW (2021). Association between the traffic level from other areas and the COVID-19 prevalence at the provincial levels in South Korea. Int. J. Infect. Dis..

[17] UNWTO (2020). Impact Assessment of the COVID-19 Outbreak on International Tourism.

[18] Seoul City. *COVID-19 Dashboard*. https://www.seoul.go.kr/coronaV/coronaStatus.do (2020) [**in Korean**].

[19] Burns J (2021). International travel-related control measures to contain the COVID-19 pandemic: A rapid review. Cochrane Database Syst. Rev..

[20] Omori R, Mizumoto K, Nishiura H (2020). Ascertainment rate of novel coronavirus disease (COVID-19) in Japan. Int. J. Infect. Dis..

[21] Dickens BL (2020). Strategies at points of entry to reduce importation risk of COVID-19 cases and reopen travel. J. Travel Med..

[22] Lim J-S, Cho S-I, Ryu S, Pak S-I (2020). Interpretation of the basic and effective reproduction number. J. Prev. Med. Public Health.

[23] Jeong J, Kwon HM, Hong SH, Lee MK (2020). Estimation of reproduction number for COVID-19 in Korea. J. Korean Soc. Qual. Manag..

[24] Lauer SA (2020). The incubation period of coronavirus disease 2019 (COVID-19) from publicly reported confirmed cases: Estimation and application. Ann. Intern. Med..

[25] Infectious Diseases Society of America (2021). IDSA Guidelines on the Diagnosis of COVID-19: Molecular Diagnostic Testing.

[26] Chen C-M (2019). Evaluating the efficiency change and productivity progress of the top global telecom operators since OTT's prevalence. Telecommun. Policy.

[27] Lee S (2018). Night Owl Bus: An ICT-supported public transport option for night time workers and the young in Seoul, South Korea. Inst. Transp. Eng..

[28] Kim, M. *et al.* In *Proceedings of the 26th ACM SIGKDD International Conference on Knowledge Discovery & Data Mining* 3466–3473.

[29] Sawano T (2020). Underestimation of COVID-19 cases in Japan: An analysis of RT-PCR testing for COVID-19 among 47 prefectures in Japan. QJM.

[30] Wu SL (2020). Substantial underestimation of SARS-CoV-2 infection in the United States. Nat. Commun..

[31] Hale T, Webster S (2020). Oxford COVID-19 government response tracker. Nat. Hum. Behav..

[32] Nishiura H (2020). The rate of underascertainment of novel coronavirus (2019-nCoV) infection: Estimation using Japanese passengers data on evacuation flights. J. Clin. Med..

[33] He X (2020). Temporal dynamics in viral shedding and transmissibility of COVID-19. Nat. Med..

[34] Jung S-M (2020). Real-time estimation of the risk of death from novel coronavirus (COVID-19) infection: Inference using exported cases. J. Clin. Med..

[35] McAloon C (2020). Incubation period of COVID-19: A rapid systematic review and meta-analysis of observational research. BMJ Open.

[36] Shearer F (2020). Assessing the risk of spread of COVID-19 to the Asia Pacific region. medRxiv.

