# Supplementary material for: A measure to estimate the risk of imported COVID-19 cases and its application for evaluating travel-related control measures
Source: Sci Rep. 2022 Jun 9;12:9497. doi: 10.1038/s41598-022-13775-0 (PMC9178220; doi:10.1038/s41598-022-13775-0)
Supplement: Supplementary file 1 — Supplementary Information. [file 41598_2022_13775_MOESM1_ESM.pdf]

SUPPLEMENTARY FILE

Title: A measure to estimate the risk of imported COVID-19 cases and its application for evaluating travel-related control measures

Authors: Heewon Kang, Kyung-Duk Min, Seonghee Jeon, Ju-Yeun Lee, Sung-il Cho

1. Weekly average travel volume to Seoul and the COVID-19 prevalence per 100,000 for each country

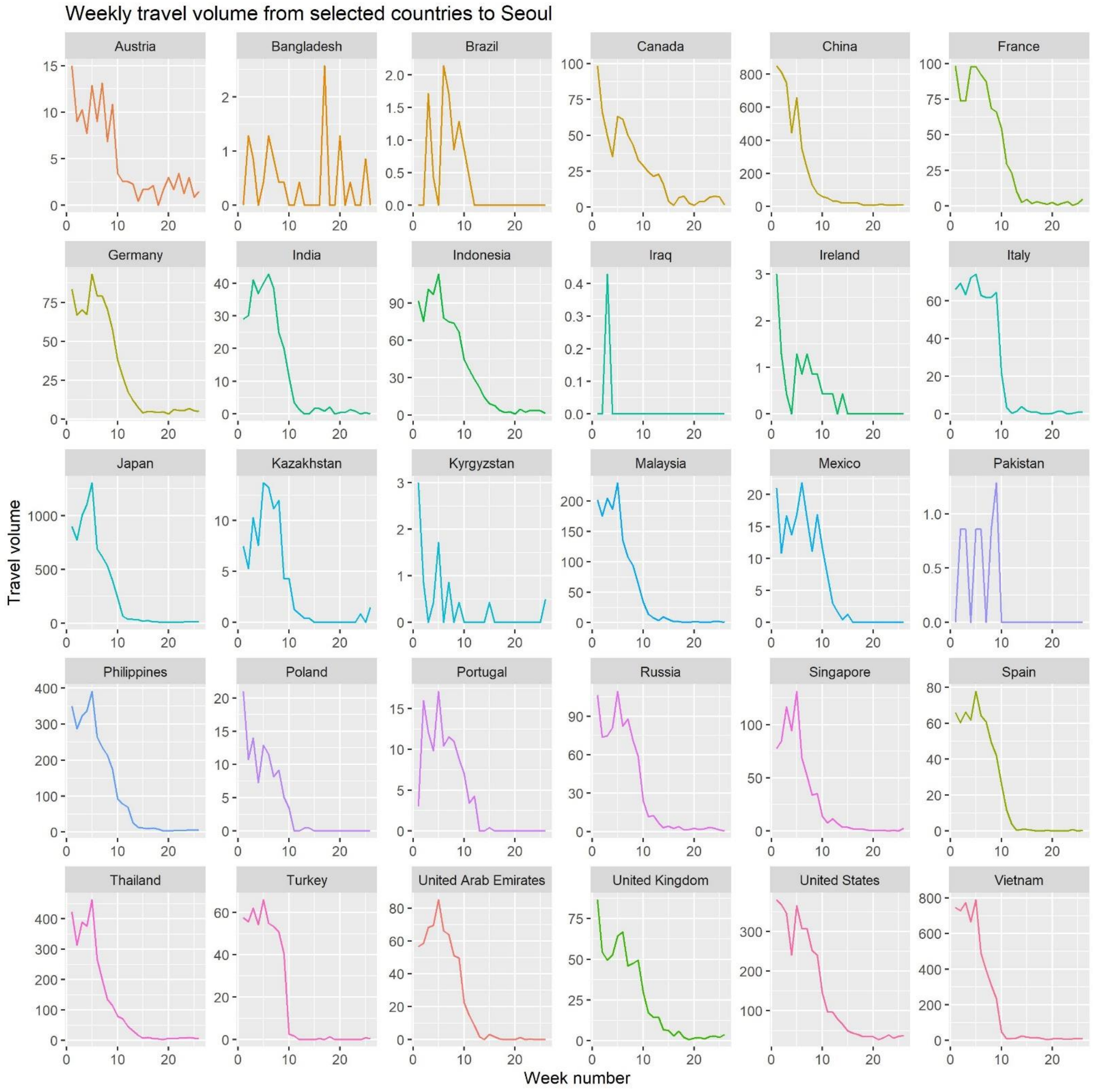

Figure S1 Weekly travel volume from selected countries to Seoul

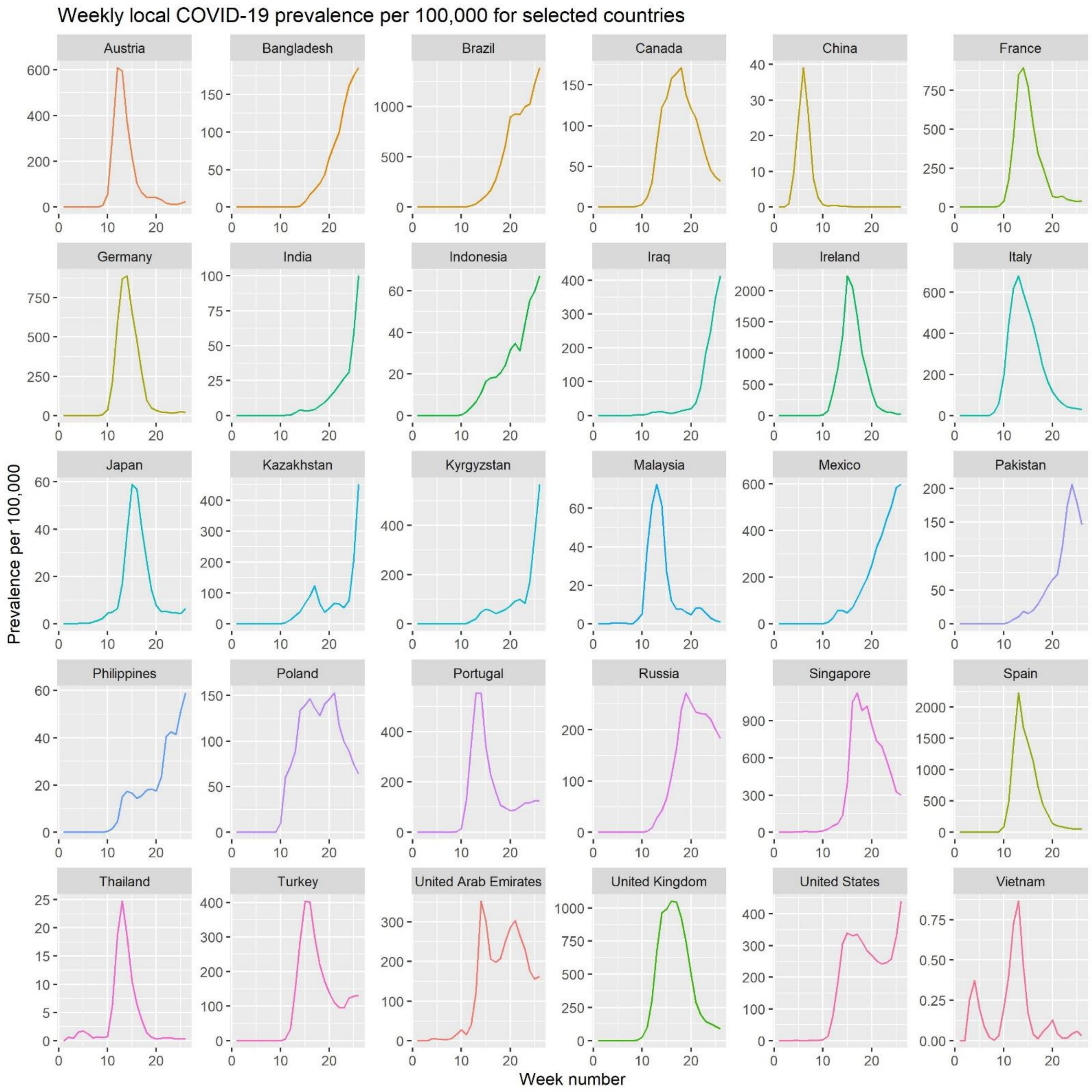

Figure S2 Weekly COVID-19 prevalence per 100,000 for selected countries

2. Expected and reported imported COVID-19 cases in Seoul during weeks 16–26 (2020.04.13–2020.06.28)

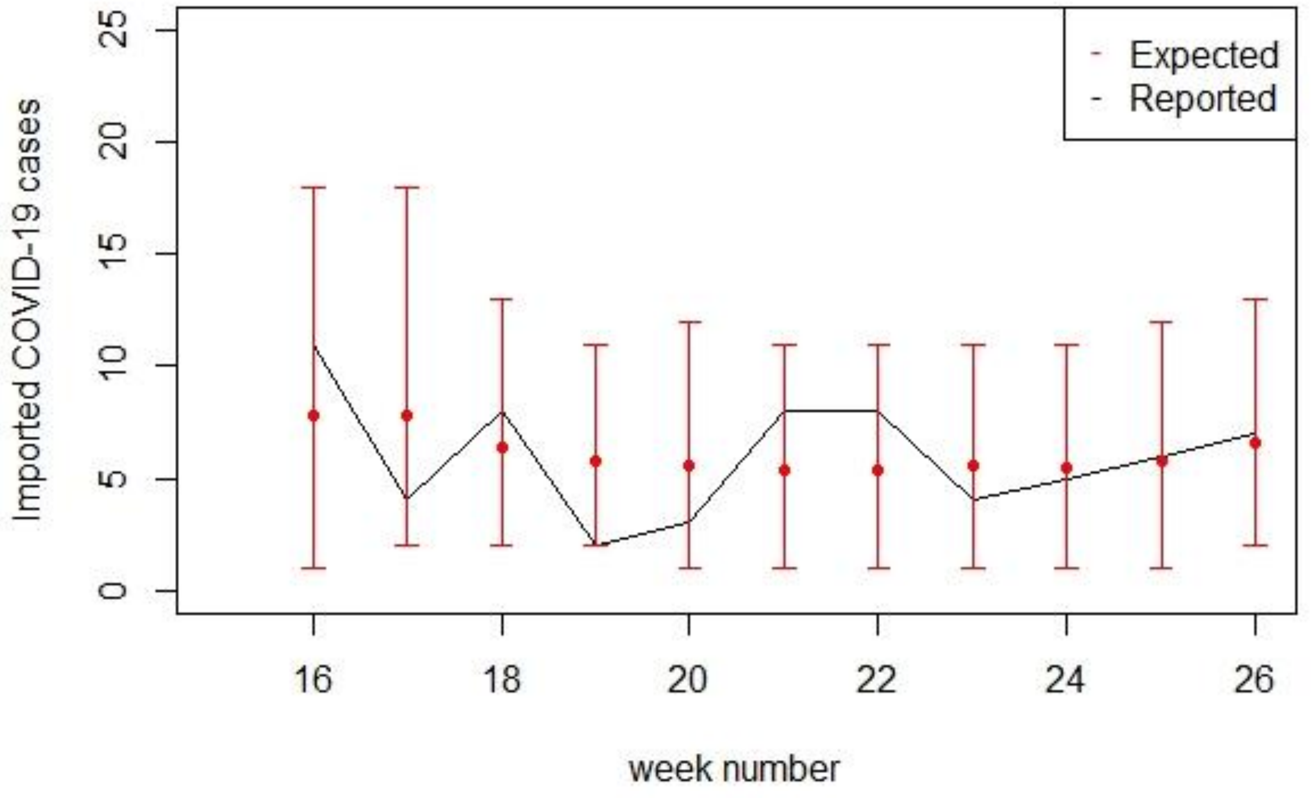

Figure S3 Expected and reported imported COVID-19 cases in Seoul

3. Sensitivity analyses with different reporting rate assumptions

In addition to the reporting rates suggested by Nishiura et al.,[1] we used two additional studies[2,3] to test the impact of different reporting rate assumptions. Reporting rate estimates from these studies considered multiple nations. Lau et al.[2] reported the reporting rates of eight epicenters: China (5%), South Korea (22%), Japan (8%), Italy (2%), France (10%), Spain (7%), Iran (4%), and the United States of America (1%). We assigned testing policy strength 0 to the lowest reporting rate, 1%. Testing policies 1, 2, and 3 were assigned to the second lowest reporting rate (2%), average rate of the eight countries (7%), and highest reporting rate (22%), respectively. Phipps et al.3 calculated reporting rates in two different time periods. The detection rate was estimated as 2.6% (1.5–3.8%) in March 2020 and 16.1% (9.2–23.1%) in August 2020. We assigned the lower (1.5%) and upper (3.8%) bounds of the detection rate in March as testing policy strength 0 and 1, respectively, and lower (9.2%) and upper (23.1%) bounds of the detection rate in August as testing policy strengths 2 and 3. Figures S4 and S5 show COVID-19 prevalence with different detection rate assumptions. Based on the prevalence calculated, we estimated the number of expected imported cases (Table S1).

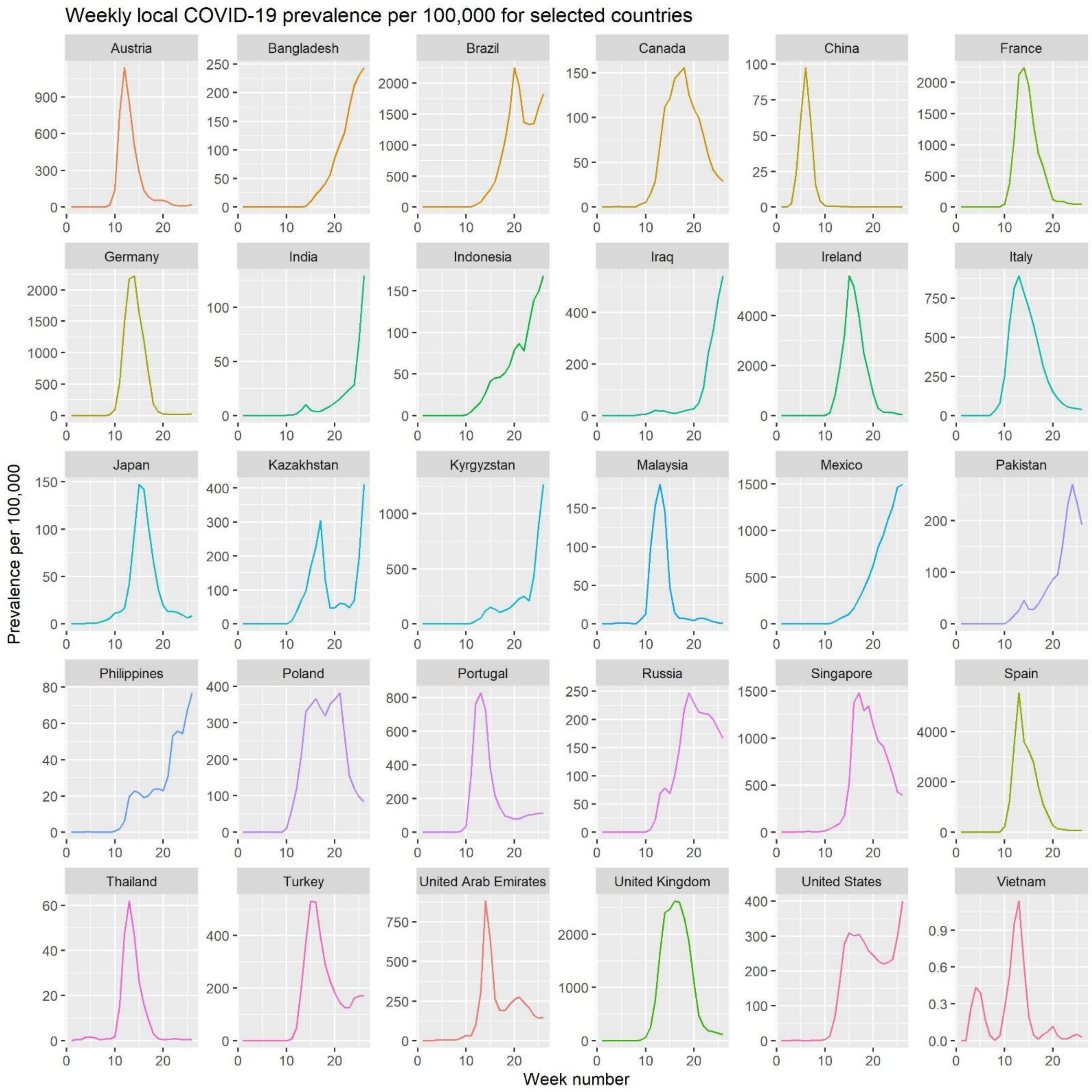

Figure S4 Weekly travel volume from selected countries to Seoul (derived based on reporting rates in Lau et al. 2021)

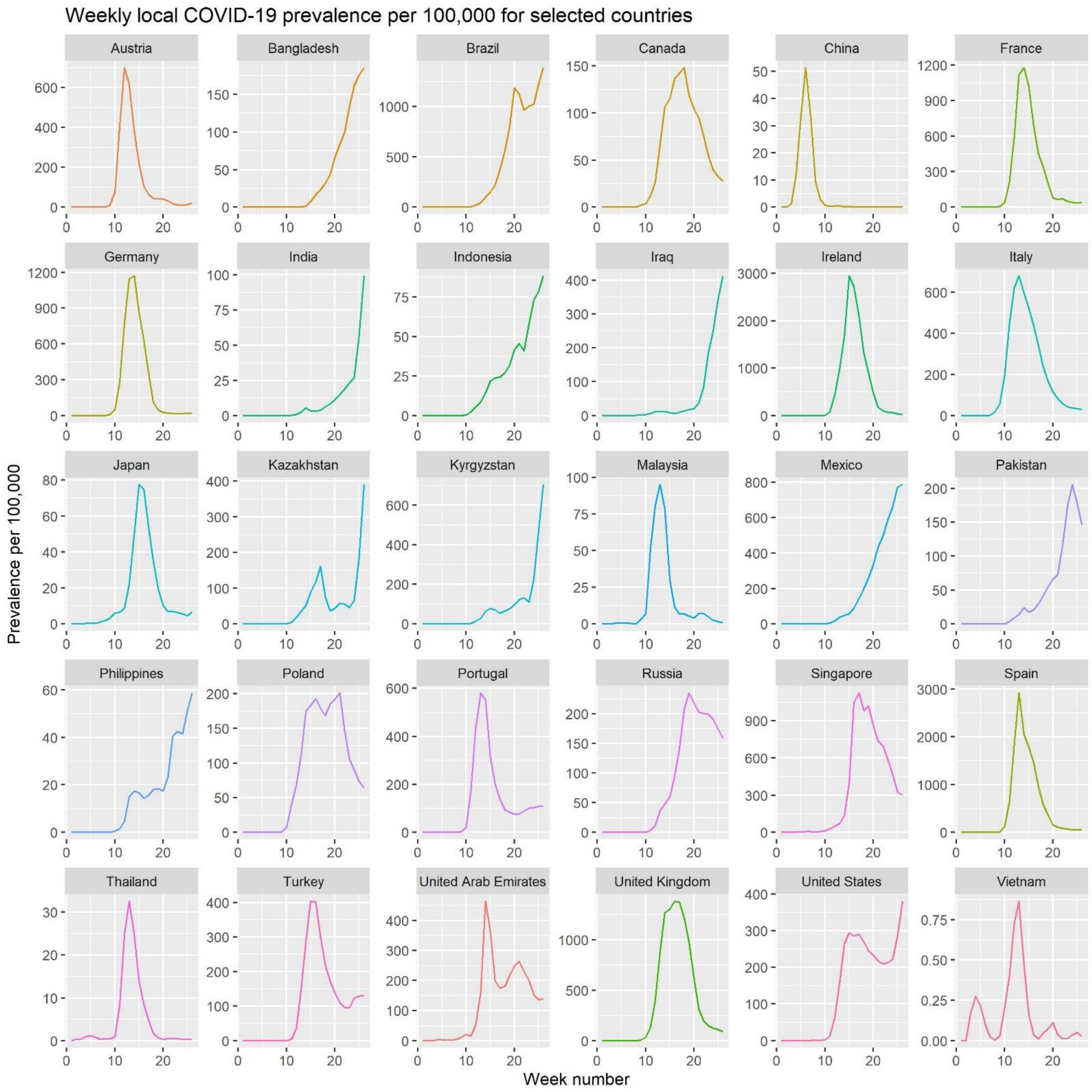

Figure S5 Weekly travel volume from selected countries to Seoul (derived based on reporting rates in Phipps et al., 2021)

Table S1 Reported imported cases and expected imported cases based on different detection rate assumptions

Week number (Date)	Reported imported cases[a]	Expected imported cases (95% CI) – Nishiura	Expected imported cases (95% CI) – Lau	Expected imported cases (95% CI) – Phipps
1 (2020-01-01 - 2020-01-05)	0	4.3 (0-12)	4.9 (1-11)	4.5 (0-12)
2 (2020-01-06 - 2020-01-12)	0	4.4 (0-11)	4.9 (1-12)	4.6 (1-12)
3 (2020-01-13 - 2020-01-19)	0	4.5 (1-12)	5.0 (1-11)	4.7 (1-11)
4 (2020-01-20 - 2020-01-26)	1	4.9 (1-11)	5.6 (1-11)	5.2 (1-11)
5 (2020-01-27 - 2020-02-02)	3	6.5 (1-12)	8.3 (1-22)	7.2 (1-15)
6 (2020-02-03 - 2020-02-09)	3	6.0 (1-11)	7.4 (1-17)	6.6 (2-13)
7 (2020-02-10 - 2020-02-16)	0	5.1 (1-11)	5.9 (2-11)	5.4 (1-11)
8 (2020-02-17 - 2020-02-23)	1	4.7 (1-12)	5.3 (1-11)	4.9 (1-11)
9 (2020-02-24 - 2020-03-01)	4	5.1 (1-10)	5.7 (1-11)	5.3 (1-10)
10 (2020-03-02 - 2020-03-08)	0	5.8 (1-11)	6.6 (1-13)	6.2 (2-12)
11 (2020-03-09 - 2020-03-15)	5	7.4 (1-15)	9.3 (1-29)	8.2 (1-21)
12 (2020-03-16 - 2020-03-22)	22	11.5 (1-60)	15.3 (0-163)	13.3 (0-101)
13 (2020-03-23 - 2020-03-29)	64	13.2 (0-92)	15.8 (0-174)	14.5 (0-131)
**Total/Average**[c]	103	83.3 (10-280)	99.9 (12-496)	90.7 (13-371)
**R^2**		0.75	0.59	0.69

[a] Indicates cases from the selected 23 countries

REFERENCES

1 Nishiura, H. *et al.* The rate of underascertainment of novel coronavirus (2019-nCoV) infection: estimation using Japanese passengers data on evacuation flights. (2020).

2 Lau, H. *et al.* Evaluating the massive underreporting and undertesting of COVID-19 cases in multiple global epicenters. **27**, 110-115 (2021).

3 Phipps, S. J., Grafton, R. Q. & Kompas, T. J. R. S. o. s. Robust estimates of the true (population) infection rate for COVID-19: a backcasting approach. **7**, 200909 (2020).
